# Toxins from the Caribbean sea anemone Bunodeopsis globulifera increase cisplatin-induced cytotoxicity of lung adenocarcinoma cells

**DOI:** 10.1186/1678-9199-19-12

**Published:** 2013-05-07

**Authors:** Heidi I Monroy-Estrada, Yolanda I Chirino, Irma E Soria-Mercado, Judith Sánchez-Rodríguez

**Affiliations:** 1 Facultad de Ciencias Marinas, Universidad Autónoma de Baja California, Ensenada, Baja California State, Mexico; 2 Unidad de Biomedicina, Facultad de Estudios Superiores Iztacala, Universidad Nacional Autónoma de México, Tlalnepantla, Estado de México, Mexico; 3 Unidad Académica de Sistemas Arrecifales, Puerto Morelos, Instituto de Ciencias del Mar y Limnología, Universidad Nacional Autónoma de México, Puerto Morelos, Quintana Roo State, Mexico

**Keywords:** Cnidaria, Pharmacology, Human lung cancer cells, Cytotoxicity assay, Cisplatin efficacy

## Abstract

**Background:**

Lung cancer causes 1.4 million deaths worldwide while non-small-cell lung cancer (NSCLC) represents 80-85% of the cases. Cisplatin is a standard chemotherapy against this type of cancer; however, tumor cell resistance to this drug limits its efficacy. Sea anemones produce compounds with pharmacological activities that may be useful for augmenting cisplatin efficacy. This study aimed to evaluate the pharmacological activities of crude venom (CV) from the sea anemone *Bunodeopsis globulifera* and four derived fractions (F1, F2, F3 and F4) to test their increase efficiency cisplatin cytotoxicity in human lung adenocarcinoma cells.

**Results:**

Pre-exposure to CV, F1 and F2 fractions increases cisplatin cytotoxicity in human lung adenocarcinoma cells under specific conditions. Exposure to CV at 50 μgmL^-1^ induced a reduction of approximately 50% in cell viability, while a similar cytotoxic effect was observed when cell culture was exposed to F1 at 25 μgmL ^-1^ or F2 at 50 μgmL^-1^. The cell culture exposure to F1 (10 μgmL^-1^) fraction combined with cisplatine (25 μM) provoked a decrease in MTT reduction until 65.57% while F2 (25 μgmL^-1^) fraction combined with cisplatin (10 μM) provoked a decrease in MTT reduction of 72.55%.

**Conclusions:**

The F1 fraction had the greatest effect on the lung adenocarcinoma cell line compared with CV and F2. The combination of antineoplastic drugs and sea anemone toxins might allow a reduction of chemotherapeutic doses and thus mitigate side effects.

## Background

According to the International Agency for Research on Cancer (IARC) and World Health Organization, cancer is a leading cause of death worldwide, accounting for 7.6 million deaths (around 13% of all deaths) in 2008. Lung cancer causes 1.4 million deaths, followed by stomach (740,000 deaths), liver (700,000 deaths), colorectal (610,000 deaths) and breast cancer (460,000 deaths). Around 70% of all cancer deaths occurred in low- and middle-income countries. Projections to 2030, indicate about 11 million of cancer deaths worldwide and it is expected that non-small-cell lung cancer (NSCLC) will represent 80-85% of cases.

NSCLC, which comprises adenocarcinoma, squamous cell carcinoma and large-cell carcinoma, is an exclusion diagnosis [1]. Platinum-based chemotherapeutics are the most common regimens for NSCLC patients but resistance and side effects have limited their efficiency [2-4]. In this regard, *Cis*-diammine-dicholoplatinium (II), known as cisplatin, is an antineoplastic drug widely used in the treatment of many solid tumors, including lung, head, neck, ovary, breast, colorectal and cervical cancers [5,6]. However, the usage of cisplatin in cancer treatment is limited due to resistance and side effects including nephrotoxicity [7].

The abovementioned problem has led to the development of new strategies to achieve effectiveness against cancer. In this sense, the peptides and proteins found in marine venoms may increase the efficacy of conventional chemotherapeutic drugs [8]. In particular, sea anemones produce peptides and proteins that act as cytolysins which are classified based on their molecular weight as follows: 5-8 kDa peptides with antihistamine activity; ~20 kDa pore-forming proteins; ~30-40 kDa cytolysins with or without phospholipase A_2_ activity; and a putative protein group ~80 kDa [9].

An essential feature of cytolysins is their ability to form pores in biological membranes, which lead to osmotic changes and loss of intracellular metabolites thereby provoking cell death [10,11]. However, cytolysins may induce cell damage in both normal and cancer cells if attached to their membrane. Nevertheless, characteristics of cancer cells are different from normal cells, including gene profile expression, metabolism and membrane features. In this regard, it has been demonstrated that cytolysins can induce more damage in cancer cells due to their differences from normal cells. For this reason, cytolysins have been proposed as an alternative for cancer therapy [8,12,13].

For example, actinoporins isolated from the tropical sea anemone *Heteractis crispa* have shown anticancer properties through the induction of p53-independent apoptosis [14]. *Bunodeopsis globulifera* – found in the reef lagoon at Puerto Morelos, Quintana Roo, Mexico – has been reported as producing a highly dangerous sting, probably due to the toxins it contains [15]. However, the potential benefits *of B. globulifera* have been not been documented. In the present work we hypothesized that toxins from *B. globulifera* could increase cytotoxicity induced by an antineoplastic agent against cancer cells. Specifically, we are interested in lung cancer cells because the incidence and mortality rates are among the highest in the world. This study aimed to investigate the ability of crude venom (CV) and fractions extracted from the sea anemone *B. globulifera* to increase the cisplatin-induced cytotoxicity in lung adenocarcinoma cells. Our results showed for first time the ability of *Bunodeopsis globulifera* CV and fractions to increase the cytotoxic effect of a common antineoplastic agent used in several types of cancer.

At this point it should be mentioned that toxins from *Bunodeopsis globulifera* are cytotoxic like other cnidarian venoms. However, the purpose of this work is to test whether the usage of low concentrations of toxins can increase the effect of a low concentration of a common antineoplasic agent. The general objective of this research is to design a strategy to decrease the concentrations of cisplatin and toxins, since this could be helpful in avoiding the side effects of higher concentrations. For example, cisplatin is useful to treat lung cancer but provokes nephrotoxicity as its main side effect. The combination of two drugs may facilitate the diminution of the side effects associated with higher doses.

## Methods

### Reagents

F-12K Nutrient Mixture medium was purchased from Gibco Invitrogen (USA). RPMI-1640 medium was obtained from In Vitro, S.A (Mexico). Fetal bovine serum (FBS) was from PAA Laboratories (Canada). Cisplatin (cat. n. P4394) and 3-(4,5-dimethylthiazol-2-yl)- 2,5-diphenyltetrazolium bromide (MTT; cat. n. M2128) were purchased from Sigma Chemical Co. (USA). Lactate dehydrogenase kit (cat. n. 630117) was obtained from Clontech Laboratories, Inc (USA). Quick Start Bradford Protein Assay Kit (cat. n. 500-0202) was bought from Bio-Rad Laboratories, Inc (USA). Sephadex G-50 Medium Gel (cat. n. 17-0043-02) was obtained from Pharmacia Biotech (Sweden).

### Cell cultures

Human A549 non-small-cell lung cancer line, which is an adenocarcinoma epithelial-derived cell line, was obtained from the American Type Culture Collection (ATCC, USA).

Cells were cultured in monolayers at 37°C in a humidified atmosphere of 5% CO_2_ in F12K medium supplemented with 10% heat-inactivated FBS.

### Isolation and Pre-purification of crude venom

Specimens of *Bunodepsis globulifera* were collected from the reef lagoon at Puerto Morelos, Quintana Roo, Mexico, by scuba diving between February and June, 2009 and from May to June, 2011. The sea anemones were identified by Ricardo Gonzalez-Muñoz. The crude venom was obtained by mincing and homogenizing the whole body of the sea anemone, and then centrifuging at 3200 g for 15 minutes at 4°C, after which the supernatant was lyophilized. The crude lyophilized venom (4 g) was purified by Sephadex G-50 M gel filtration column (85 × 5 cm) equilibrated with 0.3 M acetic acid buffer and eluted with the same buffer at a flow rate of 2 mL min^-1^. The four fractions obtained were concentrated and lyophilized.

### Cytotoxicity assays

Lung adenocarcinoma cells (1 × 10^4^ per well/150 μL) were incubated in 96-well plates and cultured for 24 hours to allow adherence. After this time, cell culture was exposed to the following treatments: 1, 10, 25, 50, 75, 100 and 200 μgmL^-1^; and 10 and 25 μgmL^-1^ of CV, BgG50F1 and BgG50F2 fractions of *B. globulifera* for 24 hours, 37°C and 5% of CO_2_ atmosphere. After each treatment, the cells were exposed to cisplatin 10 μM or cisplatin 25 μM for 24 hours.

### Cell viability assay and cytotoxicity

Cell viability was measured by the reduction of MTT to formazan blue. Cell culture was incubated with 0.5 mg/mL MTT at 37°C in an atmosphere of 5% CO_2_ for 2 hours. The medium was removed and the formazan blue crystals were dissolved in isopropyl alcohol for measuring the optical density at 540 nm [16].

The cytotoxicity was measured using a lactate dehydrogenase (LDH) cytotoxicity detection kit according to the manufacturer’s protocol. Briefly, supernatants from cell treatments were collected and diluted 1:3 with PBS. After that, supernatant dilution was mixed 1:1 with dye solution and incubated at 37°C for 30 minutes under agitation; and absorbance was measured at 540 nm.

### Quantification of protein

The protein content was measured by the Bradford assay [17] using bovine gamma globulin (BGG) as standard.

### SDS-PAGE electrophoresis

The obtained fractions were assessed by SDS-PAGE according to Laemmli [18] using a 12% polyacrylamide gel for the molecular weight determination.

### Statistical analysis

Data were expressed by mean ± SD, followed by one-way analysis of variance (ANOVA) with Bonferroni’s multiple comparison tests, performed using GraphPad Prism for Windows (GraphPad Software, USA). *p* < 0.001 was considered to be statistically significant.

## Results

### Pre-purification of crude venom from *B. Globulifera*

**Figure 1:**
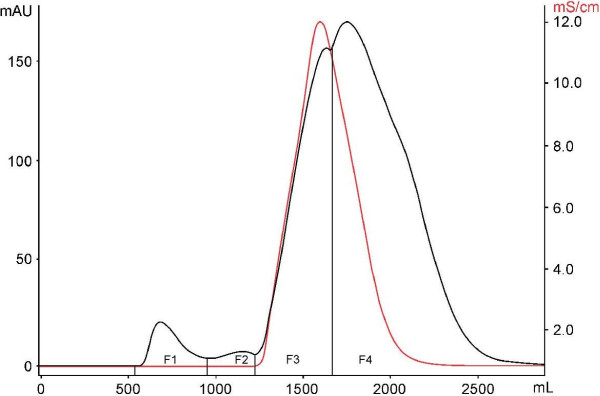
**Gel filtration chromatography Sephadex G-50 M of CV (4 g) from *B. globulifera*.** The column was equilibrated and eluted with 0.3 M acetic acid buffer at a flow rate of 2 mL min^-1^ to obtain the fractions F1, F2, F3 and F4.

Crude venom (CV) of *B. globulifera* was obtained by mincing and homogenizing the whole body of the sea anemone. It was centrifuged at 3200 *g* for 15 minutes at 4°C, and the supernatant was lyophilized. Crude venom was purified by gel filtration in a Sephadex G-50 M column and eluted with 0.3 M acetic acid buffer at a flow rate of 2 mL min^-1^. Four fractions – F1, F2, F3 and F4 – were obtained. Thereafter, fractions were vacuum-concentrated and lyophilized (Figure 1). Table 1 shows the protein content of CV, F1, F2, F3 and F4 quantified by the Bradford method [17].

**Table 1:** **Protein quantification of CV and fractions isolated from *B. globulifera *at 1 mgmL^-1^ using the Bradford method**

**Sea anemone toxins**	**Protein (μg)**
Crude venom	32.4
Fraction F1	157.7
Fraction F2	152.6
Fraction F3	20.4
Fraction F4	19.7

**Figure 2:**
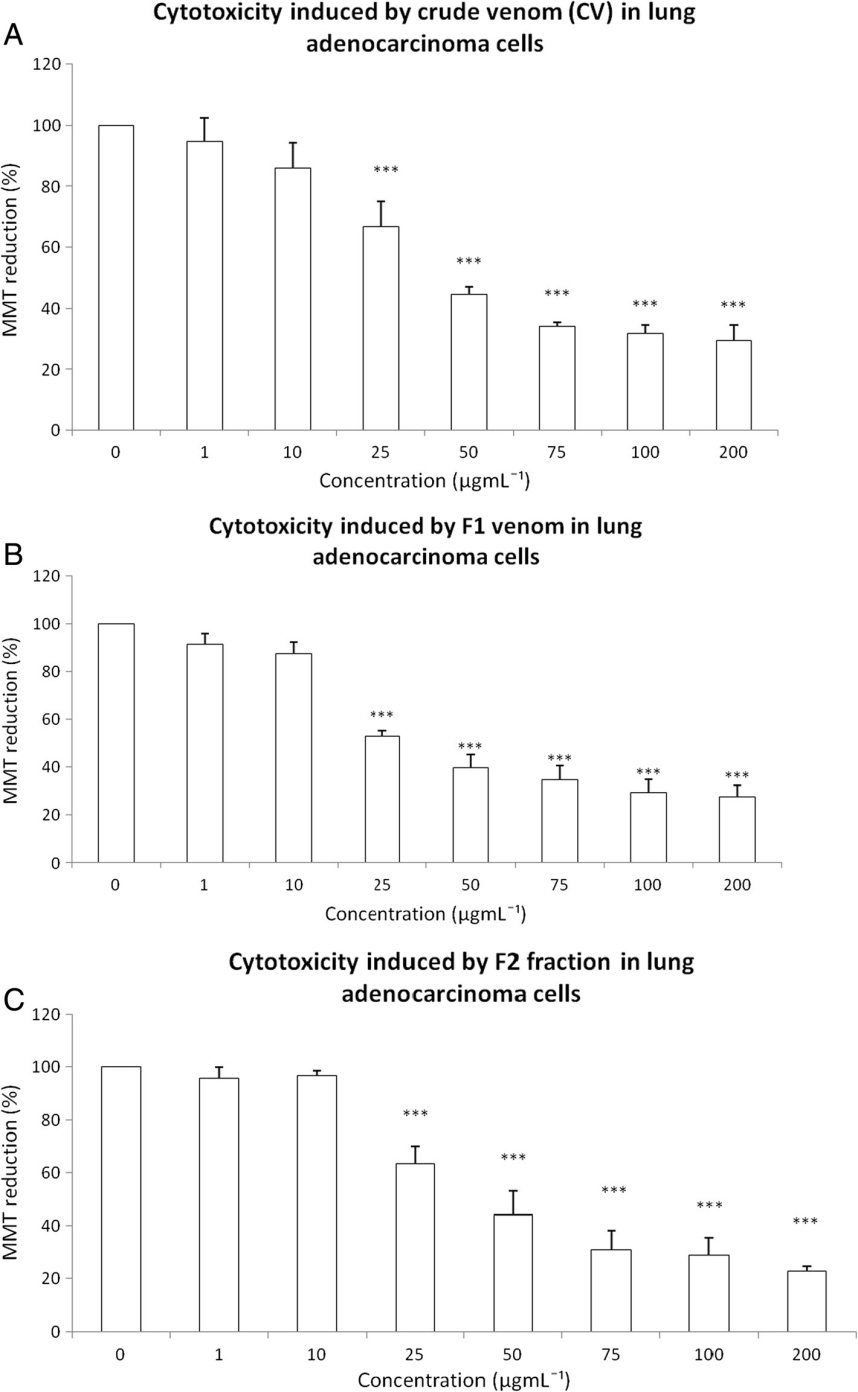
**Cytotoxicity induced by CV and the fractions F1 and F2 from *B. globulifera *at the concentrations 1, 10, 25, 50, 75, 100 and 200 μgmL^-1^ on lung adenocarcinoma cell culture.** Effect of: (**A**) CV, (**B**) F1 fraction, (**C**) F2 fraction. Graphs show the percentage of MTT reduction. Data are expressed as the mean ± SD of four independent experiments. Significance was defined as *** *p* < 0.001 (ANOVA, Bonferroni’s post test) in comparison with the untreated control values (100%).

The cytotoxic effects of CV as well as the F1, F2, F3 and F4 fractions were tested on a human lung adenocarcinoma cell line using an MTT assay. The exposure to 1 μgmL^-1^ or 10 μgmL^-1^ to CV did not induce changes in MTT reduction. However, CV at 25 μgmL^-1^, 50 μgmL^-1^, 75 μgmL^-1^, 100 μgmL^-1^ and 200 μgmL^-1^ decreased cell viability by the following percentages: 33.37, 55.46, 65.8, 68.36 and 70.44, respectively (Figure 2A). The exposure to F1 at 1 μgmL^-1^ or 10 μgmL^-1^ did not show a cytotoxic effect; however, 25 μgmL^-1^, 50 μgmL^-1^, 75 μgmL^-1^, 100 μgmL^-1^ and 200 μgmL^-1^ induced respective decreases in cell viability of 47.11%, 60.21%, 65.27%, 70.87% and 72.35% (Figure 2B). The exposure to F2 at 1 μgmL^-1^ or 10 μgmL^-1^ did not produce any change in MTT reduction, but 50 μgmL^-1^, 75 μgmL^-1^, 100 μgmL^-1^ and 200 μgmL^-1^ induced cell-viability diminutions of 36.49%, 55.83%, 69%, 71.27% and 77.13%, respectively (Figure 2C). The F3 and F4 did not alter MTT reduction (data not shown) and thus were not considered for further experiments in the present work.

### Inhibitory concentration (IC50) values for cisplatin

**Figure 3:**
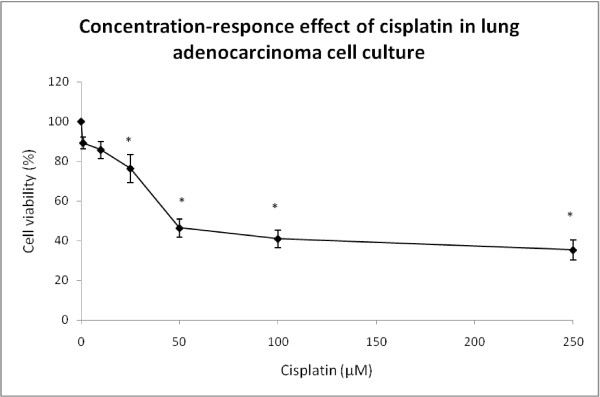
**Concentration-response effect of cisplatin on lung adenocarcinoma cell culture: cell viability was measured by MTT assay.** Data are expressed as the mean ± SD of four independent experiments. *p < 0.01 vs. control, one-way analysis of variance, with Bonferroni’s test.

The cisplatin concentration that inhibited cell growth by 50% (IC50) was determined in a human lung adenocarcinoma cell line. A cisplatin concentration between 1 and 250 μM was used in cell culture for 24 hours in three independent experiments. After treatment, cytotoxicity was tested by MTT reduction and the IC50 found to be 50 μM (Figure 3). However, concentrations below IC50 were used to test whether CV and its fractions could enhance the cisplatin cytotoxicity. Human lung adenocarcinoma cells exposed to cisplatin at 1 and 10 μM remained without changes in MTT reduction; however, cisplatin concentrations of 25, 50, 100 and 250 μM induced respective cell-viability decreases of 23.64%, 53.59%, 58.98% and 64.64% (Figure 3). We selected cisplatin concentrations of 10 μM and 25 μM, which are below the IC50.

### CV, F1 and F2 enhanced cisplatin-induced cytotoxicity

Lung adenocarcinoma cells were pre-exposed to CV, F1 or F2 at 10 μgmL^-1^ or 25 μgmL^-1^ for 24 hours. After this time, the medium was removed and cell culture was washed and exposed to cisplatin at 10 μM or 25 μM. The abovementioned strategy was executed in order to increase cisplatin cytotoxicity by pretreatment with CV, F1 or F2 at low concentration.

**Figure 4:**
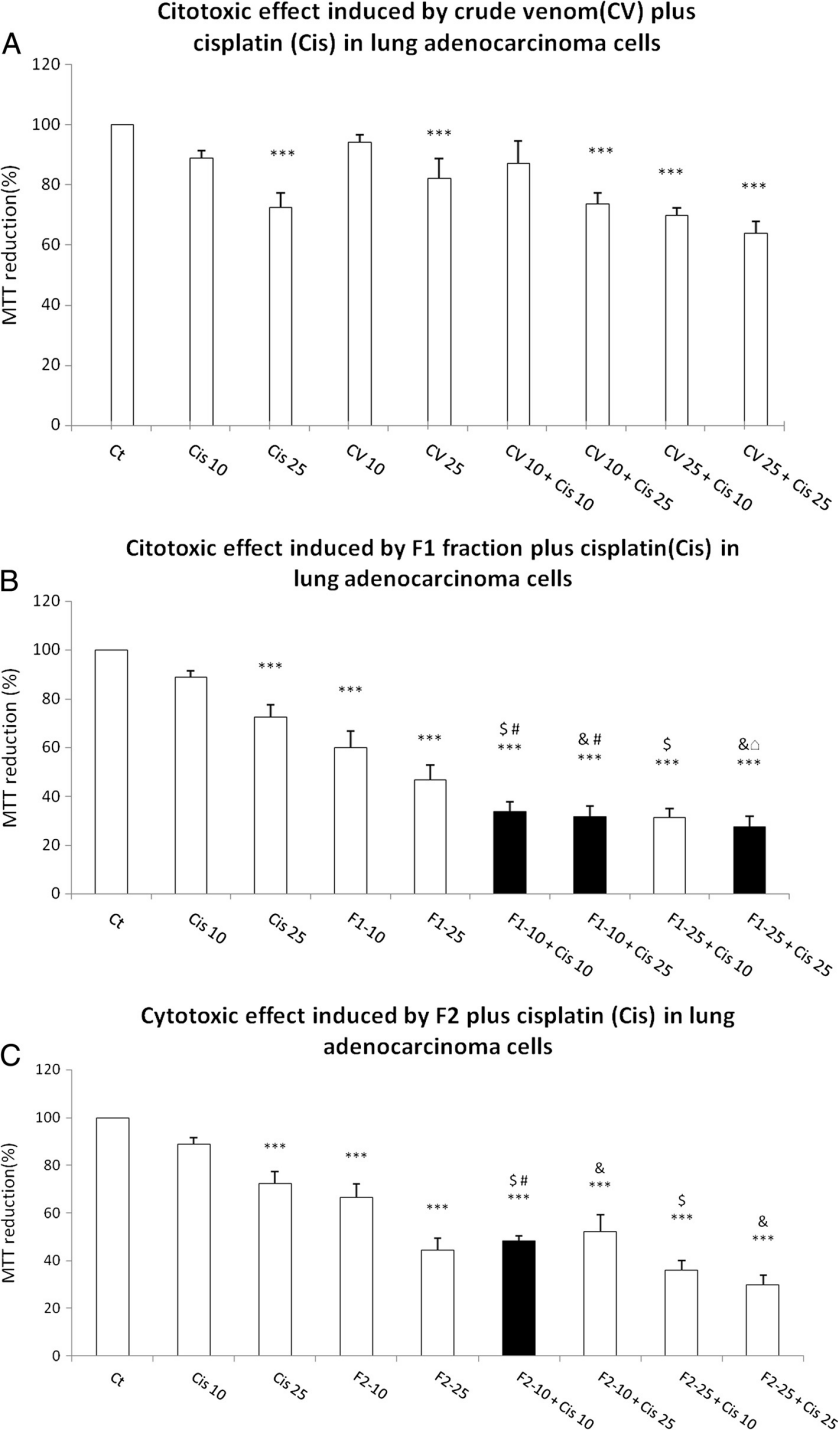
**Cytotoxic effect induced by CV, F1 and F2 fraction from *B. globulifera *on adenocarcinoma lung cell culture.** (**A**) CV, (**B**) F1 fraction plus cisplatin, (**C**) F2 fraction plus cisplatin. Graphs show the percentage of MTT reduction and data are expressed as the mean ± SD of four independent experiments. ***p < 0.001 vs. control, ^$^*p* < 0.001 vs. Cis 10, ^&^*p* < 0.001 vs. Cis 25, ^#^*p* < 0.001 vs. CV 10 or F1-10 or F2-10, ^□^*p* < 0.001 vs. CV 25 or F1-25 or F2-25 (One-way analysis of variance plus Bonferroni’s test). Gray bars show the ability of (**B**) F1 and (**C**) F2 to augment the cisplatin decrease in MTT reduction.

The pretreatment with CV at 10 μgmL^-1^ plus cisplatin at 25 μM yielded a 26.47% decrease in cell viability; the pre-treatment with 25 μgmL^-1^ CV plus 10 μM cisplatin augmented the cell-viability decrease to 30.22%. But the maximum effect was produced by 25 μgmL^-1^ CV plus 25 μM cisplatin (Figure 4A). However, the pre-exposure to CV did not increase the cisplatin-induced cytotoxicity (Figure 4A).

The pre-exposure to F1 at 10 μgmL^-1^ caused a decrease in MTT reduction induced by 10 μM and 25 μM of cisplatin to 66.39% and 68.4%, respectively (Figure 4B). The pre-exposure to 25 μgmL^-1^ F1 had similar effect; it reduced cell viability down to 72.55% after exposure to 25 μM cisplatin (Figure 4B). The 10 μgmL^-1^ F2 increased the cytotoxicity induced by 10 μM cisplatin to 51.56% (Figure 4C).

**Figure 5:**
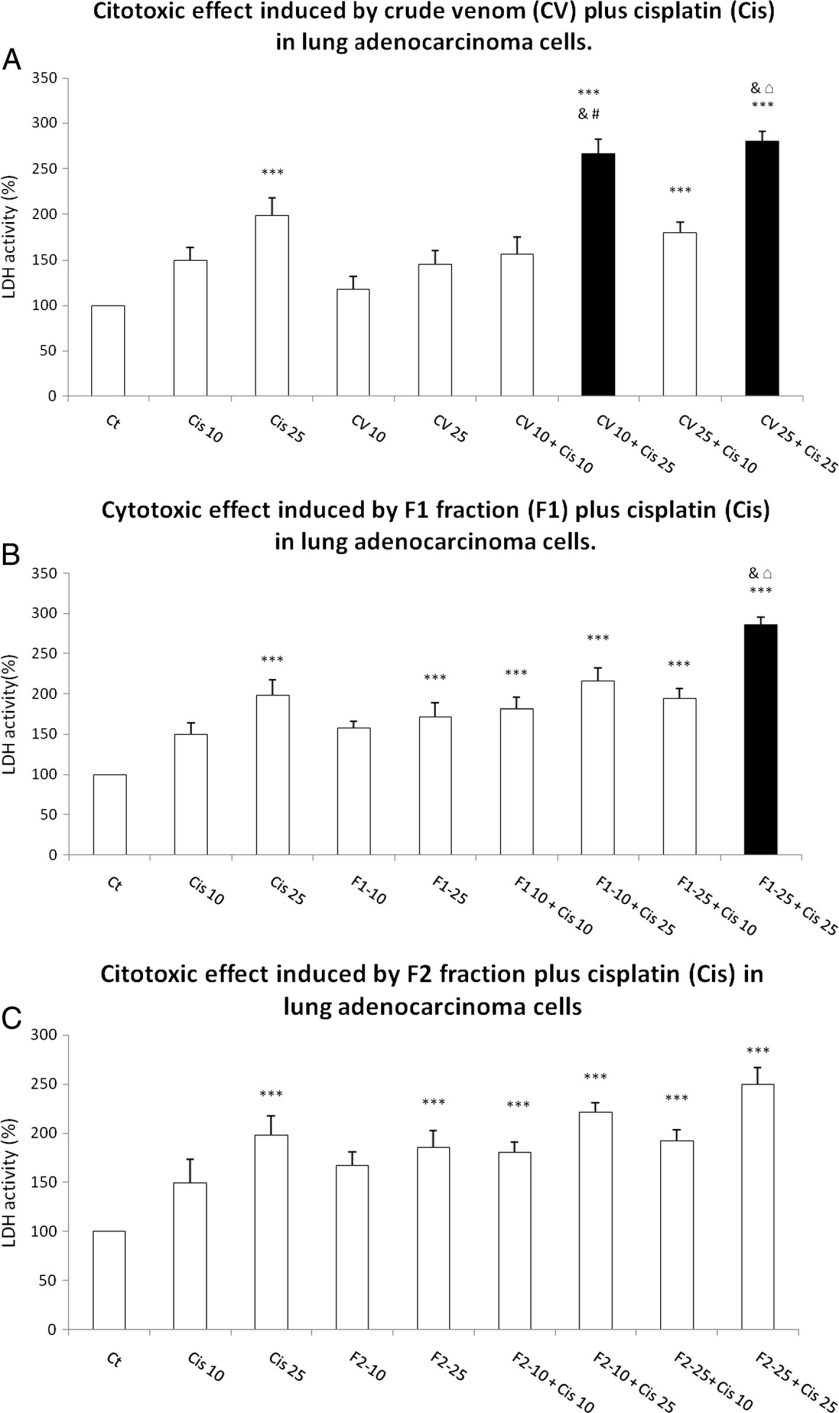
**Cytotoxic effect induced by CV, F1 and F2 fraction from *B. globulifera *on adenocarcinoma lung cell culture: (A) CV, (B) F1 fraction plus cisplatin, (C) F2 fraction plus cisplatin.** Graphs show the percentage of LDH release, with data expressed as the mean ± SD of four independent experiments. ***p < 0.001 vs. control, ^$^*p* < 0.001 vs. Cis 10, ^&^*p* < 0.001 vs. Cis 25, ^#^*p* < 0.001 vs. CV 10 or F1-10 or F2-10, ^□^*p* < 0.001 vs. CV 25 or F1-25 or F2-25 (one-way ANOVA, Bonferroni’s post test) **(a)** CV plus cis, **(b)** F1 plus cis, **(c)** F2 plus Cis. Gray bars showed the ability of (**B**) F1 and (**C**) F2 to increase the cisplatin-induced LDH release.

Then, while evaluating the LDH release into the cell culture medium as a loss of membrane integrity, we observed that 10 μgmL^-1^ CV plus 25 μM cisplatin and 25 μgmL^-1^ CV plus 25 μM cisplatin induced significant increases of 2.665- and 2.799-fold in LDH release, respectively (Figure 5A). The pre-exposure to F1 at 25 μgmL^-1^ plus cisplatin at 25 μM produced a significant 2.858 fold increase in LDH release (Figure 5B). Pre-exposure to 10 and 25 μgmL^-1^ F2 showed no statistical increase in cytotoxicity induced by 25 and 10 μM cisplatin (Figure 5C).

## Discussion

The present work showed that CV of *B. globulifera* and F1 and F2 obtained from pre-purification by Sephadex G-50 M gel filtration chromatography induced cytotoxicity in a human lung adenocarcinoma cell line. Exposure to CV at 50 μgmL^-1^ induced a reduction of approximately 50% in cell viability, while a similar cytotoxic effect was observed when cell culture was exposed to F1 at 25 μgmL^-1^ or F2 at 50 μgmL^-1^.

This cytotoxic effect has been also presented by other sea anemones, such as crude venoms of *Heteractis magnifica, Stichodactyla haddoni* and *Parachondylactis sinensis* on mouse fibroblast cell line L929 and leukemia cell line P388. In those studies, the decrease in cell viability was concentration-dependent [19]. EqTX-II cytolysin toxin from the sea anemone *Actinia equina* also induces a decrease in the viability of V-79-379 A cells (diploid lung fibroblast from Chinese Hamster) in a concentration-dependent manner [20]. RTX-A cytolysin from the sea anemone *Heteractis crispa* reduced cell viability of JB6 P^+^ Cl41 cells, Hela, THP-1, MDA-MB-231 and snu-c4 human tumor cell lines [14].

These results have shown that sea anemone toxins and some proteins derived from them exert cytotoxic activity on a non-small lung cancer cell lines, although the cytotoxic mechanisms induced by the sea anemone toxins have been sparsely investigated. In this regard, Soletti *et al.*[8] showed that the cytolysins EqTx-II and Bc2 from sea anemones potentiated the cytotoxicity induced by low-dose concentrations of the chemotherapeutic drugs against human glioblastoma cells through necrosis-like cell death [8].

In the present work, it has been observed that CV, F1 and F2 fractions from *B. globulifera* increase the cytotoxic effect induced by cisplatin under specific conditions. The exposure to F1 at 10 μgmL^-1^ induced a decrease of 40.02% in MTT reduction versus 11.12% by cisplatin. However, the pre-exposure to F1 (10 μgmL^-1^) provoked a decrease in MTT reduction of 66.30% (Figure 4B). The combination of F1 (10 μgmL^-1^) and 25 μM cisplatin decreased the MTT reduction to 67.57%, which may indicate the absence of a difference between 10 μM and 25 μM cisplatin (Figure 4B). The exposure to 25 μgmL^-1^ F1 induced a decrease of 53.42% in MTT reduction but in combination with 25 μM cisplatin, which by itself induced a decrease of 25.55% in MTT, there was a decrease of 72.55% (Figure 4B).

A similar effect was observed when lung adenocarcinoma cell line was exposed to 25 μgmL^-1^ F2 followed by 10 μM cisplatin, which induced a decrease of 51.56% in MTT reduction, while 25 μgmL^-1^ F2 induced only a decrease of 33.48% and 10 μM cisplatin a decrease of 11.12% in MTT reduction (Figure 4C).

LDH activity was measured in a cell culture medium, since this enzyme is released under conditions of cell damage, wherein we observed that 10 μgmL^-1^ CV induced an increase of 17.5% of activity versus 98% by 25 μM cisplatin; the combination induced 166.59% of increase (Figure 5A). The augmentation of LDH activity was 45% after exposure solely to CV at 25 μgmL^-1^, but rose to 1423.3% when this CV was combined with 25 μM cisplatin (Figure 5A). This finding may signify the absence of a real difference between 10 and 25 μgmL^-1^ CV. Similarly, the 71.6% increase in LDH activity produced by treating cell culture with only F1 at 25 μgmL^-1^ rose to 185.8% when the fraction was combined with 25 μM cisplatin (Figure 5B).

Based on the results of the present work, we suggest not only that CV, F1 and F2 may present different mechanisms of cell damage in lung adenocarcinoma cells, but also that CV may augment cell damage by compromising the membrane, given the evident changes in LDH release (Figure 5A), and F2 fraction, which could cause higher mitochondrial damage, since MTT reduction is related to mitochondrial activity (Figure 5C). However, F1 fraction may produce greater mitochondrial alterations and membrane damage (Figures 4B and 5B) than CV and F2 fraction.

Interestingly, this study proposes the usage of cisplatin at a dose below IC_50_, which in turn might represent fewer side effects. It is also interesting that cisplatin causes hardly any mitochondrial damage below 25 μM; however, the F1 and F2 fractions caused more damage, as measured by MTT reduction, than cisplatin alone while their combination seems to produce an additive effect (Figure 4B and C). It is also compelling that the combination of CV and F1 fraction with cisplatin appears to have synergistic effect, specifically in LDH release (Figure 5A and B).

Based on the data of the present work, we suggest that more studies are needed to investigate the cytotoxicity mechanism of CV, F1 and F2, which could help to design a combinatory treatment to increase the effects of antineoplasic agents, including cisplatin. We also suggest that these compounds may execute their toxicity though a different cellular mechanism that includes mitochondrial damage and alterations of the cell membrane.

## Conclusions

We observed that CV of *B. globulifera* and its F1 and F2 fractions obtained by Sephadex G-50 M gel filtration have a cytotoxic effect on the A549 lung cancer cell line. The F1 fraction had the greatest effect compared with CV and F2. The combination of antineoplastic drugs and sea anemone toxins might allow a reduction of chemotherapeutic doses and thus mitigate side effects. It will be necessary to evaluate not only the damage and action mechanisms in lung adenocarcinoma cells, but also whether these compounds are cytotoxic to different types of lung cancer cells and other cancer cell lines, including breast, cervix and colon cancers, each of which has a high incidence and prevalence.

## Competing interests

The authors declare that there are no conflicts of interest.

## Authors’ contributions

HIME performed experiments; YIC designed experiments and drafted the manuscript; IESM designed experiments and drafted the manuscript; JSR conceived, designed, coordinate the study and drafted the manuscript. All authors read and approved the final manuscript.
